# Recent advances in managing a spinal cord injury secondary to trauma

**DOI:** 10.12688/f1000research.7586.1

**Published:** 2016-05-27

**Authors:** Christopher S. Ahuja, Allan R. Martin, Michael Fehlings

**Affiliations:** 1 Division of Neurosurgery, Department of Surgery, University of Toronto, Toronto, Ontario, Canada; 2 Institute of Medical Science, University of Toronto, Toronto, Ontario, Canada; 3 McEwen Centre for Regenerative Medicine, UHN, University of Toronto, Toronto, Ontario, Canada; 4 Department of Surgery, University of Toronto, Toronto, Ontario, Canada; 5 Spine Program, University of Toronto, Toronto, Ontario, Canada; 6 McLaughlin Center in Molecular Medicine, University of Toronto, Toronto, Ontario, Canada

**Keywords:** Spinal cord injury, trauma, MRI, imaging, stem cell, neuroprotection, neuroregeneration

## Abstract

Traumatic spinal cord injuries (SCIs) affect 1.3 million North Americans, producing devastating physical, social, and vocational impairment. Pathophysiologically, the initial mechanical trauma is followed by a significant secondary injury which includes local ischemia, pro-apoptotic signaling, release of cytotoxic factors, and inflammatory cell infiltration. Expedient delivery of medical and surgical care during this critical period can improve long-term functional outcomes, engendering the concept of “Time is Spine”. We emphasize the importance of expeditious care while outlining the initial clinical and radiographic assessment of patients. Key evidence-based early interventions (surgical decompression, blood pressure augmentation, and methylprednisolone) are also reviewed, including findings of the landmark Surgical Timing in Acute Spinal Cord Injury Study (STASCIS). We then describe other neuroprotective approaches on the edge of translation such as the sodium-channel blocker riluzole, the anti-inflammatory minocycline, and therapeutic hypothermia. We also review promising neuroregenerative therapies that are likely to influence management practices over the next decade including chondroitinase, Rho-ROCK pathway inhibition, and bioengineered strategies. The importance of emerging neural stem cell therapies to remyelinate denuded axons and regenerate neural circuits is also discussed. Finally, we outline future directions for research and patient care.

## Introduction

Traumatic spinal cord injuries (SCIs) have devastating consequences for patients and families. Direct lifetime costs can be as high as $1.1–$4.6 million per patient, with over 1.3 million patients affected in North America alone ^1, 2^. Expedient delivery of specialized medical and surgical care can improve long-term functional outcomes for patients ^3, 4^. The rapidly evolving management of patients with SCI is summarized here with emphasis on evidence-based current practices and upcoming therapies in trial.

## Pathophysiology

**Figure 1.**
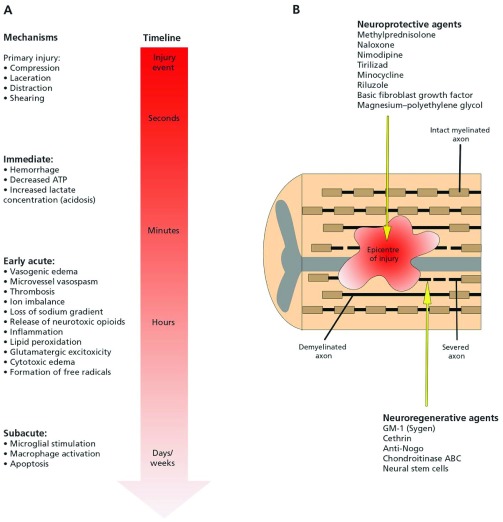
“( **A**) Primary and secondary mechanisms of injury determining the final extent of spinal cord damage. The primary injury event starts a pathobiological cascade of secondary injury mechanisms that unfold in different phases within seconds of the primary trauma and continuing for several weeks thereafter. ( **B**) Longitudinal section of the spinal cord after injury. The epicenter of the injury progressively expands after the primary trauma as a consequence of secondary injury events. This expansion causes an increased region of tissue cavitation and, ultimately, worsened long-term outcomes. Within and adjacent to the injury epicenter are severed and demyelinated axons. The neuroprotective agents listed act to subvert specific secondary injuries and prevent neural damage, while the neuroregenerative agents act to promote axonal regrowth once damage has occurred. ATP = adenosine triphosphate.” Reprinted with permission from Wilson J, Forgione N, Fehlings MG. Emerging therapies for acute traumatic spinal cord injury. *CMAJ*. 2012; 185(6): 485 ^4^.

The initial primary trauma results in mechanical injury to cells, damages the sensitive microvasculature of the cord, and causes hemorrhage. Pro-apoptotic signaling is initiated and progressive edema contributes to ongoing ischemia ^5, 6^. Furthermore, the blood-spinal cord barrier is disrupted, permitting an influx of vasoactive peptides, cytokines, and inflammatory cells ^7, 8^. Over the ensuing hours to days, by-products of cellular necrosis are released (ATP, DNA, and K ^+^), creating a cytotoxic post-injury milieu and activating microglia to further recruit phagocytes. Macrophages and polymorphonuclear leukocytes infiltrate and generate oxygen free radicals and other cytotoxic by-products. Excess glutamate release and failure of reuptake by astrocytes results in excitotoxicity for adjacent neurons ^9, 10^. Please see Figure 1.

As the parenchymal volume is lost, cystic cavities coalesce, creating a physical barrier to cell migration ^11^. Furthermore, the lack of structural framework impedes regenerative attempts. Over time, astrocytes proliferate and surround the perilesional zone, creating an irregular mesh-like barrier of interwoven cell processes ^12^. This is accompanied by fibroblast deposition of chondroitin sulfate proteoglycans (CSPGs) including neural/glial antigen 2 (NG2) and tenascin ^13– 16^. CSPGs and myelin glycoproteins act via the Rho-ROCK (rho-associated protein kinase) pathway to inhibit neurite outgrowth by signaling growth cone collapse through effector kinases ^17^. Together, these mechanisms severely restrict endogenous neural circuit regeneration and oligodendrocyte remyelination at a cellular level.

Systemically, cervical and thoracic cord injuries can interrupt the sympathetic output of the intermediolateral column, causing neurogenic shock with loss of peripheral vascular tone and bradycardia ^18^. The result can be profound hypotension, which further exacerbates cord ischemia ^19^. Paralysis of the intercostal and abdominal muscles restricts the inspiratory phase of ventilation, leading to hypercarbia and/or hypoxia. Furthermore, a weakened cough, poor mobilization, and secondary immunodeficiency (immune paralysis) after SCI make patients highly susceptible to life-threatening infections ^20, 21^.

There is currently a lack of consensus on the optimal approach to several areas of SCI diagnosis and treatment, in part owing to heterogeneity in injuries (cervical versus thoracic, complete versus incomplete) but also owing to conflicting interpretations of the literature. As discussed below, early recognition and appropriate triage of patients are critical first-line components of care; however, the choice of imaging modalities for diagnosis and prognostication remains unclear ^22^. Care is largely supportive, but the long-term importance of early surgical decompression (<24 hours) ^3, 23, 24^, blood pressure augmentation (mean arterial pressure [MAP] ≥85 mmHg) ^4^, and selective use of methylprednisolone (MPSS) ^25– 28^ is increasingly being recognized. Even under ideal circumstances, recovery of lost function is patient dependent and largely determined by their clinical status at 1–2 years post-injury. Below we summarize the current standards of care and discuss recent advances in the diagnosis, neuroprotection, prognostication, and regeneration for patients with SCI.

## Current care

The first-line care of a patient with SCI involves securing the airway, breathing, and circulation followed by appropriate spinal immobilization in the field to limit further insult of the highly susceptible cord during transport ^22^. Recognition and appropriate triage of SCI patients is critical in the early period to ensure timely delivery of interventions at specialized centers ^22^. While maintaining spinal immobilization, airway and breathing management can range from supplemental oxygen to intubation and ventilation. At any point during the acute injury, systemic hypotension (systolic blood pressure [SBP] <90 mmHg) is associated with worse neurologic outcomes ^22^. With a profound loss of vascular tone and potential bradycardia, patients can rapidly fall into neurogenic shock. Large-volume intravenous (IV) fluid therapy (most often crystalloids) is the mainstay of treatment; however, adjunctive alpha-adrenergic vasopressors (e.g. norepinephrine and phenylephrine) may also be used to temporize patients. As soon as the patient is resuscitated, an American Spinal Injury Association (ASIA) International Standards for Neurological Classification of SCI (ISNCSCI) examination should be completed to establish the level of injury and baseline function ^22^.

Early imaging to localize and classify the injury is critical to expeditiously manage patients and provide the outcome-altering early interventions discussed below ^3, 4, 23, 24^. CT is recommended in all patients, as plain radiographs can miss 6% of injuries ^29^. In those with cervical injuries and high-energy mechanisms, imaging of the thoracolumbar spine is also recommended to detect concomitant injuries ^30^. Magnetic resonance imaging (MRI) can be useful to assess ligamentous injury, critical disc herniations, and epidural hematomas and enhance prognostication of outcomes ^31^; however, its role in the initial workup of patients with SCI remains unclear given resource constraints at many centers. Urgent MRI is recommended by the authors in cases with unexplained neurological deficits to ensure that ongoing cord compression or ligamentous injuries are not missed.

While establishing the diagnosis and classifying the injury pattern, secondary injuries should be avoided by transferring the patient to an intensive care unit (ICU) setting with respiratory, hemodynamic, and cardiac monitoring ^22^. Polytrauma patients should continue to have acute life- or limb-threatening injuries managed while maintaining appropriate spinal immobilization and recognizing early therapeutic windows for SCI interventions. This requires a concerted interdisciplinary effort including modified surgical positioning for orthopedic/general surgery procedures, fiberoptic tracheal intubation, and clear communication between teams.

### Early surgical decompression

Progressive edema and hemorrhage contribute to ongoing mechanical pressure on the sensitive microvascular circulation. Surgical decompression aims to relieve this pressure to reduce secondary ischemic-hypoxic injury. The Surgical Timing in Acute Spinal Cord Injury Study (STASCIS) was a prospective, observational study of 313 patients with cervical SCI. Patients undergoing early decompression (<24 hours from injury; mean = 14 hours) were more than twice as likely to have a two-grade ASIA Impairment Scale (AIS) improvement at 6-month follow-up than those undergoing late surgery (≥24 hours from injury; mean = 48.3 hours) ^23^. No difference in complication rates between early (24%) and late (30%) surgery was found (p = 0.21). These findings were further confirmed in a prospective Canadian cohort study ^24^. Another observational study reported shorter hospital lengths of stay (LOS) for ASIA grade A (complete) or grade B (incomplete sensory injury; complete motor injury) patients undergoing early decompression ^3^. An additional multi-center, European (SCI-POEM) study is currently underway ^31^. The main critique of these studies is their cohort design, which was chosen for both practical reasons and ethical concerns surrounding randomizing patients where true clinical equipoise does not exist. However, these studies represent the most robust, large-scale data on surgery for SCI and provide support for a well-studied intervention in a field where few treatment options exist for patients. Given this data, the concept of “Time is Spine” has emerged, emphasizing the critical importance of early therapies to improve long-term functional outcomes ^4^. Furthermore, early surgical decompression (<24 hours) is recommended in current American Association of Neurological Surgeons (AANS) and Congress of Neurological Surgeons (CNS) guidelines ^22^.

### Mean arterial pressure

To further mitigate ischemia of the injured cord, blood pressure augmentation has emerged as a viable strategy. Maintaining MAP ≥85–90 mmHg post-injury has been shown to improve AIS grade outcomes for patients ^4^. Current AANS/CNS guidelines provide level III recommendations to maintain MAP for 7 days post-injury. This requires maintenance of a euvolemic or slightly hypervolemic state using IV crystalloid in addition to an infusion of vasopressors and invasive blood pressure monitoring (e.g. arterial line). These significant requirements have led to a non-inferiority trial of MAP ≥65 mmHg versus MAP ≥85 mmHg called MAPS, which is expected to be completed in 2017 ^31^.

Maintaining these MAP parameters can also be a barrier to mobilization, which is critical in the early post-injury period to prevent complications. Safely elevating patients and engaging muscle activity requires a collaborative, multidisciplinary effort along with adjuncts such as prophylactic vasopressors and peripheral/abdominal binding. The ideal time to begin mobilization should be evaluated on an individual basis according to the patient’s hemodynamics, underlying comorbidities, and the expertise of the healthcare team.

### Early intravenous methylprednisolone

MPSS is a synthetic corticosteroid which upregulates anti-inflammatory factors and decreases oxidative stress to enhance endogenous cell survival in animal models of SCI. A series of key clinical trials entitled National Spinal Cord Injury Study (NASCIS) I (1984) ^25^, II (1990) ^26^, and III (1997) ^27^ demonstrated serious adverse events with a high-dose MPSS protocol (e.g. sepsis), which outweighed the potential benefit for neurologic recovery. However, when a low-dose protocol was given to patients within 8 hours of injury, no adverse events and a potential improvement were seen. The study methodology and subgroup analyses from this series have been extensively debated over the last several decades. To settle this debate, a comprehensive Cochrane review was published in 2012 encompassing six randomized controlled trials (RCTs) and several key observational studies. The analysis demonstrated a four-point ASIA motor score improvement for patients receiving MPSS within 8 hours of injury ^33^. While this appears to be a small improvement in relative terms, a four-point improvement in key myotomes such as grip, triceps, and deltoid function can represent a significant functional gain for patients. The 2016 AOSpine guidelines, developed by an international and interdisciplinary committee of experts, will suggest IV MPSS (administered over 24 hours) as a treatment option when feasible to patients within 8 hours of injury.

## Frontiers of imaging

Conventional MRI, producing T1- and T2-weighted images, has been shown to be of modest value in helping to predict neurological and functional outcomes, particularly the prognostic factors of length of hemorrhage and degree of cord compression ^31^. However, conventional MRI fails to yield information about the health of the spinal cord tissue as signal changes are non-specific and can reflect a range of physiological processes such as hemorrhage (macroscopic or microscopic), edema, gliosis, cell loss, and cavitation ^31^. A number of emerging MRI techniques have the potential to substantially improve our ability for prognostication by quantifying the degree of tissue injury and measuring functional changes within the spinal cord ^34^. Techniques that can quantify aspects of tissue microstructure include diffusion tensor imaging (DTI), reflecting axonal integrity, magnetization transfer (MT) and myelin water fraction (MWF), correlating with myelin quantity, and MR spectroscopy (MRS), measuring the concentration of key molecules that reflect cell loss ( *N*-acetylaspartate), gliosis (myo-inositol), and ischemia (lactate). Functional MRI (fMRI) can visualize neuronal activity and connectivity. All of these techniques are under intense investigation, with DTI in particular showing strong correlation with tissue injury, which may lead to clinical translation in the near future ^35^.

## Frontiers of neuroprotection

Neuroprotective interventions to preserve injured tissue and reduce secondary insult are key approaches in SCI. Multiple therapies targeting components of the pathophysiologic cascade are currently under investigation and in trial.

Hypothermia decreases the basal metabolic rate of central nervous system tissue and reduces inflammation ^36^. Therapeutic hypothermia (32–34°C) has been applied in the neuroprotection of patients after cardiac arrest ^37^ and neonatal hypoxic-ischemic encephalopathy ^38, 39^. Animal models of SCI have demonstrated significant improvements with systemic intravascular cooling ^40^, leading to a pilot study of 14 AIS grade A patients in which a trend towards neurologic improvement (43% versus 21%) was reported with no difference in complication rates ^41^. The pending Acute Rapid Cooling Therapy for Injuries of the Spinal Cord (ARCTIC) phase II/III trial looks to further assess the efficacy of this therapy ^42^.

Riluzole is a benzothiazole, voltage-gated sodium-channel blocker which indirectly decreases glutamate release and enhances reuptake ^43^. It has been used successfully to slow the progression of motor neuron loss and improve survival in patients with amyotrophic lateral sclerosis ^44^. A phase I/II clinical trial of 36 patients with SCI demonstrated 15.5-point improvements in ISNCSCI motor scores for riluzole-treated patients with a cervical level injury ^45^. A phase II/III RCT entitled “Riluzole in Spinal Cord Injury Study” (RISCIS), sponsored by AOSpine, the North American Clinical Trials Network (NACTN), the Rick Hansen Institute, and the Ontario Neurotrauma Foundation, is now underway to further assess efficacy for patients with C4-8 level injuries. The trial is expected to complete in 2018 ^32^. 

Minocycline is a tetracycline-class antibiotic with anti-inflammatory properties including inhibition of tumor necrosis factor-α (TNF-α), interleukin-1β (IL-1β), cyclooxygenase-2 (COX-2), nitric oxide synthase (NOS), and microglial activation. Preclinical models of SCI showed dramatically decreased lesion sizes and neuron loss with acute minocycline treatment ^46, 47^. In a mixed-level phase II study, cervical SCI patients (N = 25) had a 14-point ASIA motor score improvement with minocycline treatment versus placebo (p = 0.05) ^48^. This has led to a phase III trial (N = 248) of IV minocycline x 7 days versus placebo entitled “Minocycline in Acute Spinal Cord Injury” (MASC) to be completed by 2018 ^32^.

Fibroblast growth factor (FGF) is part of the family of heparin-binding proteins. It has been shown to protect against excitotoxic cell death and mitigate oxygen free radical production in animal models of SCI ^49^. SUN13837 (Asubio Pharmaceuticals Inc.) is an FGF analogue trialed in a phase I/II study which completed in 2015. Results are expected to be reported in the near future ^32^. Similarly, cytokine granulocyte-colony stimulating factor (G-CSF) has been shown to be neuroprotective in SCI by directly promoting cell survival and inhibiting TNF-α and IL-1β ^50^. Two non-randomized studies demonstrated improvements in AIS scores for patients receiving IV G-CSF ^51, 52^. A larger randomized trial is anticipated.

Finally, magnesium is a non-competitive NMDA receptor antagonist. It has been applied in the neuroprotection of multiple central nervous system disorders to decrease excitotoxicity and inhibit inflammation. When delivered with an excipient, such as polyethylene glycol (PEG), it generates stable cerebrospinal fluid levels in the therapeutic range ^53– 55^. AC105 (Acorda Therapeutics) is a Mg-PEG compound that was studied in a phase I trial concluding in February 2015 ^32^. Results are pending report.

## Frontiers of neuroregeneration

The majority of patients living with impairments from SCI are in the chronic phase of injury. Neuroregenerative strategies aiming to help these millions of patients are being developed by countless researchers worldwide. Significant therapeutic opportunities exist using endogenous and exogenous repair mechanisms with adjuncts to address barriers to recovery such as the loss of structural framework, cystic cavitation, astroglial/CSPG scarring, and inhibitory molecular signaling.

CSPGs, myelin-associated glycoproteins (MAGs), oligodendrocyte-myelin glycoprotein (OMgp), and neurite outgrowth inhibitor-A (NOGO-A) all act on receptors associated with the Rho-ROCK pathway to inhibit neurite outgrowth, thereby stemming attempts at recovery. Multiple types of drugs directed at disrupting this signaling cascade have been developed. Bioengineered monoclonal NOGO-A antibodies, given by intrathecal injection, have been shown to improve regeneration of rat and primate spinal cords ^56, 57^. A phase I study (N = 51) of ATI355 (anti-Nogo-A antibody) has been completed with results pending dissemination ^32^. Direct Rho inhibition has also been developed in the form of an intraoperatively applied epidural paste (Cethrin/VX-210; Vertex Pharmaceuticals) ^17^. A mixed cervicothoracic-level phase I/IIa study (N = 48) demonstrated significant motor improvement (18.5 ASIA motor score points) for cervical patients receiving Cethrin without any increase in complications ^58^. A further phase IIb trial is planned.

Instead of inhibiting the Rho-ROCK pathway, chondroitinase ABC (ChABC) is an enzyme which degrades CSPGs in the glial scar to effectively remove initiators of the cascade. In rodent models of SCI, intrathecal and intraparenchymal treatments with ChABC have been shown to reduce CSPGs, scar volume, and cavity volume. Electrophysiologic and behavioral improvements in motor and sensory function after ChABC treatment have also been demonstrated by a number of groups including seminal work by Bradbury *et al*. ^59– 61^. This exciting approach is being further developed with novel delivery methods and in combination with other regenerative techniques such as cell-based therapy ^49, 60, 62^. Furthermore, a humanized form of chondroitinase is being studied with a more central nervous system-specific motif.

Cell-based therapies are a rapidly evolving field of regenerative medicine. Embryonic stem cells (ESCs) and induced pluripotent stem cells (iPSCs), and their differentiated progeny, are capable of regenerating lost neural circuits, remyelinating denuded axons, modulating the inflammatory response, and modifying the microenvironment ^63– 65^. ESCs have been studied the longest but are in limited supply and their use raises complex ethical issues. The discovery of four factors capable of generating a pluripotent cell from adult somatic cells provided a limitless source of cells with the possibility of developing autologous therapies in the future ^66^. While previously unknown issues with iPSCs, such as epigenetic memory and early senescence, are being studied, these cells remain a key therapeutic strategy ^67^. Multiple studies of oligodendrocyte precursor cells, neural precursor cells, and cells to modify the microenvironment have produced recovery of function in preclinical models over the past three decades ^68– 73^. An international phase I/II trial of human central nervous system stem cell injections for cervical SCI is being conducted by Stem Cells Inc. with results expected in 2017 ^32^. A parallel thoracic injury phase I/II study, currently completing follow-ups, has shown improvements in sensation with no increase in complication rates ^74^. Another phase I trial of NSI-566 neural stem cells for thoracic injury is expected to conclude in 2016 ^32^. Ongoing studies will continue to address safety concerns and establish efficacy of this exciting therapy.

Several important parallel cell-based strategies are under investigation. Schwann cells (SCs) are able to remyelinate both peripheral nervous system (PNS) and central nervous system axons and are a key component of effective PNS regeneration. In animal models, SCs have been shown to reduce cystic cavitation, enhance tissue sparing, and promote behavioral recovery ^75^. The Miami Project to Cure Paralysis is currently recruiting patients with chronic ASIA grade A, B, and C cervical and thoracic injuries for a phase I (N = 10; NCT02354625), open-label trial of autologous SCs transplanted into the injury epicenter ^32^. The study is expected to conclude in 2018. The same team is also running a phase I study (N = 10; NCT01739023) of autologous SCs for subacute thoracic ASIA grade A SCI expected to report in 2016 ^32^.

Olfactory ensheathing cells (OECs) cover olfactory neurons in a manner similar to SCs. They are potent phagocytes capable of continuously clearing microbes and debris from the nasal mucosa while also secreting neurotrophic support factors ^76– 79^. OECs harvested from the nasal mucosa or olfactory bulb have been shown to enhance axonal regeneration and remyelination and significantly improve behavioral outcomes in animal models ^80^. Several chronic SCI trials of OECs have been completed and compiled in a recent meta-analysis (cumulative N = 1193) which demonstrated no significant increase in serious adverse events. Higher-quality studies are required moving forward to definitively establish efficacy ^81^.

Mesenchymal stem cells (MSCs) are multipotent stromal cells with the capacity to repair damaged tissues by differentiating along connective tissue lineages (e.g. chondrocytes, myocytes, osteoblasts, and adipocytes) ^82^. Furthermore, they are uniquely capable of modulating the inflammatory response both at a systemic level and within their local environment ^83– 85^. In animal models, MSCs have been shown to decrease peripheral inflammatory cell infiltration, enhance pro-survival trophic factor levels, and promote neural tissue sparing ^86, 87^. Numerous phase I and II trials studying autologous MSCs are ongoing worldwide. Pharmicell Co. is conducting a phase II/III trial (N = 32; NCT01676441) of autologous MSCs transplanted into the parenchyma and intrathecal space of patients with ASIA grade B injuries. The study is expected to conclude in 2016 ^32^. A similar class of support cells is bone marrow cells (BMCs) which, in preclinical testing, have been shown to facilitate directed axonal regrowth by producing extracellular matrix ^88^ and promoting remyelination ^89^. A phase I/II RCT (N = 21) of ASIA grade A patients administered autologous BMCs intraparenchymally or intrathecally was published in 2015. No serious adverse events were reported ^90^. A similar recent study in children with chronic SCI also showed no significant adverse events ^91^. Bioengineered strategies form an important complementary avenue of research for regeneration of the traumatically injured cord. Multiple biomaterials have been developed to fill cavitation defects and recreate the structural architecture required to promote endogenous and exogenous cell migration and survival ^92– 96^. These materials are being engineered to have a specified porosity and density, be immunologically inert, and biodegrade over time. Furthermore, many have been modified to release growth factors or immunomodulatory drugs to enhance regeneration ^95, 97, 98^. A unique class of biomaterial, self-assembling peptide hydrogels, has been designed to be injectable and assemble into nanofibrils resembling extracellular matrix when exposed to ionic or temperature changes ^68, 99^. As biochemical manufacturing and our transplant techniques are refined, biomaterials are likely to be important components of a successful regenerative therapy for SCI.

## Frontiers of rehabilitation

A critical part of any treatment for SCI is an effective rehabilitation strategy. This requires the integration of SCI-specific physiotherapy (e.g. stretching, strength training, and transferring), occupational therapy (e.g. modified activities for self-care), nursing (e.g. wound care and bowel/bladder care), psychology, speech-language pathology, and medicine. Conventional physical rehabilitation aims to reduce chronic complications (e.g. ulcers, deformity, and cardiorespiratory deconditioning) while enhancing residual function for maximal gain. Several technological adjuncts are actively being researched and integrated into long-term rehabilitation to achieve these goals including functional electrical stimulation (FES) and epidural stimulation (EDS). FES applies microcurrents to nerves and muscles to enhance motor function during rehabilitation or daily activities. Patterned FES has shown success in restoring both upper extremity (e.g. writing, eating, and self-care) and lower extremity (e.g. supported ambulation and stationary bicycle riding) function. FES has also been used to significantly improve volitional control of the bowel and bladder ^100^. In addition to immediate gains, FES may also produce long-term improvements similar to activity-based restorative therapy (ABRT) via mechanisms of neuroplasticity. Both ABRT and FES repeatedly activate preserved circuits to maintain existing connections while promoting synaptogenesis, myelination, and neurite sprouting ^100– 103^. Furthermore, during physical rehabilitation, FES augmentation can dramatically increase patients’ oxygen uptake and respiratory rate and improve their fat to muscle ratio ^104, 105^. A phase III trial (N = 84; NCT01292811) of FES for the restoration of upper limb function in tetraplegic patients with subacute cervical injuries is currently recruiting patients. This study is expected to conclude in 2018. EDS is a parallel approach using microcurrents delivered by epidural electrodes to stimulate the spinal cord and/or conus medullaris ^106^. It has been successfully used in the treatment of refractory neuropathic pain for numerous conditions (e.g. amputation, stroke, and SCI). The concept behind EDS-induced motor recovery is the enhancement of neuroplasticity by activating central circuits including the central pattern generator for locomotion (T11-L1) and cardiorespiratory circuits. Several phase I and II studies (NCT02592668, NCT02339233, and NCT02313194) are underway to explore the potential of EDS with results expected over the next 5 years ^32^.

## Looking forward

The landscape of SCI management is quickly changing as the heterogeneity of patients and long-term importance of key early interventions are increasingly being recognized. Combinatorial neuroprotective and neuroregenerative strategies are most likely to be effective moving forward given the multifaceted nature of the injury; however, this approach may require tailoring to specific patient subgroups. This necessitates a deeper understanding of SCI pathophysiology, clinical presentation, and relevant imaging, serum, and cerebrospinal fluid biomarkers ^107, 108^. While landmark studies of the past have enrolled varied groups of patients for logistical reasons, we foresee future studies stratifying patients by well-defined diagnostic criteria to elucidate subtle but prognostically important differences. The results of the above trials may become catalysts for critical changes in the current standard of care. Even small improvements in sensory or motor outcomes can have profound functional effects on patients’ vocational abilities and independence.

## Abbreviations

AANS, American Association of Neurological Surgeons; ABRT, activity-based restorative therapy; AIS, American Spinal Injury Association Impairment Scale; ASIA, American Spinal Injury Association; BMC, bone marrow cell; ChABC, chondroitinase ABC; CNS, Congress of Neurological Surgeons; CSPG, chondroitin sulfate proteoglycan; DTI, detrusor tensor imaging; EDS, epidural stimulation; ESC, embryonic stem cell; FES, functional electrical stimulation; FGF, fibroblast growth factor; G-CSF, granulocyte-colony stimulating factor; IL-1β, interleukin-1β; iPSC, induced pluripotent stem cell; ISNCSCI, International Standards for Neurological Classification of Spinal Cord Injury; IV, intravenous; MAP, mean arterial pressure; MPSS, methylprednisolone; MRI, magnetic resonance imaging; MSC, mesenchymal stem cell; NOGO-A, neurite outgrowth inhibitor-A; OEC, olfactory ensheathing cell; PEG, polyethylene glycol; PNS, peripheral nervous system; RCT, randomized controlled trial; ROCK, rho-associated protein kinase; SBP, systolic blood pressure; SC, Schwann cell; SCI, spinal cord injury; STASCIS, Surgical Timing in Acute Spinal Cord Injury Study; TNF-α, tumor necrosis factor-α.
